# INHBA Promotes the Progression of Gastric Cancer by Activating MAPK Signaling Pathway via Targeting ITGA6

**DOI:** 10.32604/or.2025.070333

**Published:** 2026-02-24

**Authors:** Guojian Zhou, Rui Zhang, Lei Nie, Yi Si, Ting Liu, Jing Wang, Shuangshuang Han, Mingda Xuan, Jia Wang, Weifang Yu

**Affiliations:** 1 Gastrointestinal Disease Diagnosis and Treatment Center, The First Hospital of Hebei Medical University, Shijiazhuang, 050000, China; 2 Gastroenterology Department, The Affiliated Hospital of Hebei University, Baoding, 071000, China; 3 Department of Infectious Diseases, The First Hospital of Hebei Medical University, Shijiazhuang, 050000, China

**Keywords:** Gastric cancer (GC), inhibin subunit beta A (INHBA), integrin alpha-6 (ITGA6), RNA-sequencing (RNA-seq), mitogen-activated protein kinase (MAPK)

## Abstract

**Objectives:**

Gastric cancer (GC) is among the most prevalent malignancies worldwide, ranking as the fifth most common cancer and the fifth leading cause of cancer-related mortality. This study intends to investigate how Inhibin subunit beta A (INHBA) promotes the progression of GC by activating the mitogen-activated protein kinase (MAPK) signaling pathway via targeting Integrin alpha-6 (ITGA6).

**Methods:**

Quantitative reverse transcription-Polymerase Chain Reaction (qRT-PCR) and Immunohistochemistry (IHC) were utilised to validate the expression levels of INHBA in GC, which were subsequently correlated with the clinicopathological factors and outcomes. Cellular and animal studies were conducted to ascertain the role of INHBA in GC. RNA-sequencing (RNA-seq) and bioinformatics analysis were used to screen for the downstream target and pathway of INHBA, with Co-immunoprecipitation (Co-IP), Co-Immunofluorescent (Co-IF), Western blot (WB) and Rescue experiments validating their mechanisms of action in GC.

**Results:**

IHC and qRT–PCR analysis confirmed that GC tissues exhibited higher INHBA expression than adjacent noncancerous tissues. This elevated INHBA expression was found to be significantly associated with the incidence of tumor lesions, lymph node metastasis, and progression to higher TNM stages. Functional experiments showed that INHBA promoted GC cell proliferation and enhanced their migration and invasion *in vitro* while inhibiting apoptosis. Animal studies results indicated that INHBA overexpression promoted tumor growth and increased tumor weight and volume. Through a series of experiments, including RNA-seq, Co-IP, Co-IF, WB, and rescue assays, this study demonstrated that INHBA promotes GC progression by targeting ITGA6 to regulate the MAPK signaling pathway.

**Conclusions:**

INHBA/ITGA6/MAPK axis can provide new insights into GC therapy. Targeted INHBA inhibition holds promise as a therapeutic approach for GC treatment.

## 1 Introduction

Gastric cancer (GC) continues to pose a global health burden and is one of the most prevalent malignancies worldwide [1]. According to recent GLOBOCAN estimates, it ranks as the fifth most common cancer and the fifth leading cause of cancer-related mortality globally, accounting for approximately 1 out of 20 new cancer cases and 1 out of 16 cancer-related mortalities annually [2]. The pathogenesis and progression of GC are mediated through a complex interplay of molecular mechanisms, gene-related factors, and environmental influences. Patients diagnosed with late-stage GC experience dismal clinical outcomes and typically exhibit a median overall survival (OS) of less than 12 months [3]. Treatment targeting genes with oncogenic alterations and related signaling pathways is going to be a crucial cancer treatment modality for the foreseeable future since cancer is a genetic disease. However, nearly all cancers are caused by multiple genetic alterations; however, only a subset of genetic alterations can specifically be targeted by drugs. The inability to target a number of key oncogenic alterations is one of the major limitations of targeted inhibitors [4]. Therefore, understanding the development of GC and identifying new gene-targeted treatments are crucial for improving patient care.

Inhibin subunit beta A (INHBA), a member of the transforming growth factor-beta (TGF-β) superfamily, serves as a heterodimeric protein composed of α and βA subunits. First identified in 1978 as a key modulator of the hypothalamic–pituitary–gonadal axis, INHBA forms a disulfide-linked homodimer called activin A [5]. Several studies reported that irregular INHBA expression exerted various biological functions in the developmental process of different tumors, including esophageal carcinoma and colorectal and breast cancers [6–8]. Studies have demonstrated that INHBA expression is correlated with cancer progression and poor prognosis. Emerging mechanistic studies have suggested that the role of INHBA in oncogenic processes involves epithelial–mesenchymal transition through the TGF-β/Smad signaling axis [9,10]. The potential of INHBA as a target for cancer treatment has also been validated, and early-phase studies yielded promising results [10]. These results indicated that INHBA is related to the onset, development, and prognosis of various cancers.

At present, studies on INHBA in GC are scarce. Elevated INHBA expression is correlated with reduced 5-year survival rates, demonstrating biomarker potential in gastric adenocarcinoma [11–13]. Several studies have shown that certain circular RNAs and transcription factors can regulate INHBA expression through various mechanisms, thereby affecting the biological function of GC cells and promoting GC progression [14,15]. Research has shown that INHBA genes promote the migration and invasion of GC cells by activating the TGF-β signaling pathway [16]. Growing evidence suggests that INHBA is a potential modulator in GC [13,17]; however, its precise oncogenic signatures in GC persist as an underdeveloped research frontier, with only studies on the TGF-β pathway addressing this target in GC models.

Therefore, this study intends to elucidate INHBA-driven oncogenesis through a specific signaling pathway and regulation of downstream effector genes. RNA-seq and bioinformatics analysis were used to conduct the downstream target and pathway of INHBA. Co-IP, Co-IF, WB and Rescue experiments were used to underlie the connection between INHBA and the downstream gene. In conclusion, we proposed an essential INHBA target associated with GC progression and further explored its regulatory mechanism. We hope the INHBA/ITGA6/MAPK axis can provide new insights for the treatment of GC.

## 2 Materials and Methods

### 2.1 Patients and Samples

In total, 50 pairs of GC and adjacent normal tissues were resected from patients at the First Hospital of Hebei Medical University. None of the patients underwent chemotherapy/radiation before surgery. Isolated specimens were snap-frozen in liquid nitrogen and stored at Hebei Medical University First Hospital Biobank. In this study, histologically confirmed gastric adenocarcinoma, including signet-ring cell carcinoma subtypes, was utilized for tumor tissue analysis. The study protocol, including patients and samples, was approved by the Ethics Committee of Hebei Medical University First Hospital and followed the principles of the Declaration of Helsinki (Approval No. S00826). The studies were conducted in accordance with the local legislation and institutional requirements. The ethics Committee review board waived the requirement of written informed consent for participation from the participants or the participants’ legal guardians/next of kin because the use of these specimens falls under the “exemption category” defined by the National Health Commission and the institutional ethics guidelines, as the research involves no intervention, no additional risk to patients, and no impact on their treatment or privacy. The specimens were originally retained for potential future research purposes with prior generic consent obtained during hospitalization.

### 2.2 Cell Culture

GC cells (NUGC-3, Hs746T) were obtained from Shanghai iCell Bioscience Inc., and human gastric mucosa cells (GES-1) and GC cells (HGC-27, AGS), which were confirmed by STR analysis, from Wuhan Pricella Biotechnology Co., Ltd. The cells were cultured in RPMI 1640 or DMEM (Gibco, Carlsbad, CA, USA) supplemented with 10% fetal bovine serum (Gibco) and 1% penicillin–streptomycin solution (Solarbio Science and Technology Co., Ltd., Beijing, China). Cells were cultured in a 37°C incubator with 5% CO_2_. Monthly mycoplasma screening was performed via PCR, and the number of cell passages was strictly limited to no more than 30.

### 2.3 Transfections, Stable Cell Lines, siRNAs, and Plasmids

Transfections were performed using Lipofectamine 3000 (Invitrogen; Carlsbad, CA, USA; Catalog number: L3000008) once the cells reached 60%–70% confluence. NUGC-3/Hs746T and HGC-27/AGS cells were transfected with four INHBA-targeting siRNA sequences (si281, si382, si499, si822) or overexpressed pcDNA3.1-INHBA plasmids (with negative controls; GenePharma Co., Ltd., Shanghai, China). To identify the most effective siRNA against ITGA6, we screened four candidates (si614, si1293, si1595, si2679) in AGS cells. This screening revealed siITGA6-614 as the most effective, demonstrating superior silencing efficiency. In rescue experiments, NUGC-3 received ITGA6 overexpression (pcDNA3.1-ITGA6) and INHBA silencing (siRNA-INHBA), whereas AGS underwent ITGA6 silencing (siRNA-ITGA6) and INHBA overexpression (pcDNA3.1-INHBA). Functional assays were conducted 48 h after transfection. For a stable clone selection, neomycin (600 μg/mL) was introduced into HGC-27 cultures 48 h after transfection of the INHBA overexpressed plasmid to generate oe-Vector and oe-INHBA isogenic cell lines (antibiotic-free background). G418 is an antibiotic that inhibits protein synthesis by blocking the extension steps in prokaryotic and eukaryotic cells. Once a monoclonal colony was established, the cells were cultured in medium supplemented with 300 μg/mL G418 (Solarbio Science and Technology Co., Ltd., Beijing, China) for 10 consecutive days to stabilize transgenic expression.

### 2.4 RNA Extraction and Quantitative Reverse Transcription Polymerase Chain Reaction (qRT–PCR)

Total RNA was isolated from cells (GES-1, HGC-27, AGS, NUGC-3 and Hs746T) and tissue samples using RNA-Easy Isolation Reagent (Vazyme Biotech Co., Ltd., Nanjing, China; Catalog number: R701-01). Reverse transcription was performed using the PrimeScript RT Reagent Kit (Takara Bio, Beijing, China; Catalog number: RR037A) to synthesize complementary DNA (cDNA). For quantitative reverse transcription–polymerase chain reaction (qRT–PCR), reactions were performed using AceQ Universal SYBR qPCR Master Mix (Vazyme). β-Actin mRNA was used as the endogenous reference for normalization. All experiments included three technical replicates per sample. Supplementary Table S1 presents the primers used. To calculate relative mRNA expression levels, cycle threshold values were analyzed using the 2^−∆∆CT^ method.

### 2.5 Western Blot Analysis and Antibodies

Protein extraction was performed from cells (GES-1, HGC-27, AGS, NUGC-3 and Hs746T) using RIPA lysis buffer (Solarbio, Beijing, China; Catalog number: R0010) mixed with protease/phosphatase inhibitor (Boster, Wuhan, China; Catalog number: AR1182) at a 100:1 ratio. The animal tumor tissues (INHBA over-expression and vector group) were washed with PBS buffer and cut into pieces and then the protein extraction process was the same of the cells. After shaking at 4°C for 30 min, the protein mixtures were centrifuged at 12,000× *g* for 10 min at 4°C. The supernatant was collected to detect protein concentrations using a protein assay kit (Solarbio, Beijing, China; Catalog number: PC0020) and the protein concentration was adjusted to an equivalent loading of total protein. 30 μg of total protein was loaded in each lane. Proteins were separated via electrophoresis on 10% SDS-polyacrylamide gels (Bio-Rad Laboratories Inc., Hercules, CA, USA) and then transferred to a polyvinylidene fluoride membrane (Merck Millipore, Billerica, MA, USA). After blocking with milk for 2 h, samples were incubated with diluted antibodies. The antibodies used were as follows: GAPDH (diluted 1:500; Goodhere-Bio, Hangzhou, China; Catalog number: AB-P-R001), INHBA (diluted 1:500; Proteintech, Wuhan, China; Catalog number: 17524-1-AP), ITGA6 (diluted 1:500; Proteintech, Catalog number: 27189-1-AP), MEK1/2 (diluted 1:1000; Proteintech; Catalog number: 11049-1-AP), phospho-MEK1 (diluted 1:1000; Proteintech; Catalog number: 28930-1-AP), ERK1/2 (diluted 1:1000; Proteintech; Catalog number: 66192-1-Ig), and phospho-ERK1/2 (diluted 1:100; Proteintech; Catalog number: 28733-1-AP). The Odyssey scanning system (LI-COR Biosciences, Lincoln, NE, USA) was used to identify immunoreactive protein bands. All experiments were performed in independent replicates.

### 2.6 Cell Proliferation Assay

For the CCK-8 assay, cells (HGC-27, AGS, NUGC-3 and Hs746T) from each group were evenly seeded in 96-well plates at a density of 2 × 10^3^ cells per well and cultured in complete medium. At 24, 48, 72, and 96 h post-seeding, 10 μL of a Cell Counting Kit-8 reagent (CCK-8; Dojindo, Tokyo, Japan; Catalog number: CK04) was added to each well. After a 2-h incubation, absorbance was measured at 450 nm using a Promega GloMax luminescence detector (Promega, Madison, WI, USA).

In the colony formation assay, cells (AGS and NUGC-3) were plated at a density of 1000 cells per well in 6-well plates and cultured in complete medium, replaced every 3 days. After 7–14 days, the cells were washed twice with 1× PBS (pH 7.2–7.4), fixed with 4% PFA for 30 min, and then stained with 0.1% crystal violet for 20 min. The cells were again washed twice with 1× PBS (pH 7.2–7.4) followed by dehydration in the air. Finally, the stained cell colonies were counted using the ImageJ software (ImageJ 1.53e, National Institutes of Health, Bethesda, MD, USA).

### 2.7 Cell Migration Assay

In the wound-healing assay, cells (HGC-27, AGS, NUGC-3 and Hs746T) from each group were seeded in six-well plates (3 × 10^5^ cells per well). Once the cells reached 100% confluence, a 200-μL pipette tip was used to draw two straight lines in each well to mimic wounds. After washing twice with 1× PBS (pH 7.2–7.4), the cells were cultured in serum-free medium. Wound images were obtained by a microscope (TE2000-U, Nikon, Tokyo, Japan) at 0 and 48 h, and the migration rate was calculated as the ratio of the gap width at 0 h to that at 48 h.

For the Transwell migration assay, 4 × 10^4^ (HGC-27, AGS) or 6 × 10^4^ (NUGC-3, Hs746T) cells in 200-μL serum-free medium were added to the upper chamber (Corning Incorporated, Corning, NY, USA), whereas 700 μL of complete medium was added to the lower chamber to induce cell migration. After a 48-h incubation, cells on the upper side of the polycarbonate membrane were removed using a cotton swab. The membrane was washed twice with 1× PBS (pH 7.2–7.4), fixed with 4% PFA for 30 min, and then stained with 0.1% crystal violet for 20 min. Excess stain was removed through PBS washing. Images of five randomly selected fields were obtained, and the stained cells were counted using the ImageJ software (ImageJ 1.53e, National Institutes of Health, Bethesda, MD, USA).

### 2.8 Cell Invasion Assay

To facilitate downward invasion, GC cells (HGC-27, AGS, NUGC-3 and Hs746T) were resuspended in 100-μL serum-free medium and seeded into the upper chamber, whereas 600-μL complete medium was added to the lower chamber for the Transwell invasion assay. All the other experimental procedures were performed according to the same protocol used in the Transwell migration assay.

### 2.9 Cell Apoptosis Assay

After 48 h of transfection, the cells (HGC-27, AGS, NUGC-3 and Hs746T) were collected from each group and stained with Annexin V-FITC/PI apoptosis assay kit (NeoBioscience, Shenzhen, China; Catalog number: FAK015.50). Every tube of cells was added to 300 μL buffer, followed by 5 μL Annexin-V-FITC and 10 μL PI stain, which was then shaken. The reaction was carried out in the dark at room temperature for 20 min. A total of 1 × 10^5^ cells were analyzed for apoptosis via flow cytometry (BD Biosciences, San Jose, CA, USA; number: 342976). HGC-27cells were transfected with INHBA over-expressed plasmid or empty plasmid as negative control. The flow cytometry Annexin-FITC/PI double staining method utilizes Annexin-V to specifically bind to phosphatidylserine that was efflorescated in the early stage of apoptosis, while PI only passes through the damaged cell membrane to mark nucleic acids. Lower left quadrant (Annexin-FITC-/PI-): indicating a living cell. Right lower quadrant (Annexin-FitC+/PI-): indicating early apoptotic cells. Upper right quadrant (Annexin-FITC+/PI+): corresponding to late apoptosis or necrotic cells. Upper left quadrant (Annexin-FITC-/PI+): mostly due to mechanical injury, cell fragments or necrotic cells. The apoptosis experimental design of other cell lines is similar to that of HGC27. The apoptotic rate was calculated using the FlowJo software (FlowJo 10.9.0, BD Biosciences, Portland, USA).

### 2.10 Animal Studies

A total of 10 male BALB/c nude mice weighing between 12 and 16 g and aged 4–5 weeks were obtained from Beijing Sibeifu Biotechnology Co., Ltd. They were randomly divided into two groups, each consisting of five animals, and housed in a pathogen-free environment with *ad libitum* access to food and water. The environment was maintained at a temperature of approximately 22°C and a 12-h light/dark cycle. After the skin of the left hind limb flank of the nude mice was disinfected, the mice were subcutaneously inoculated with stable overexpression of INHBA and negative control HGC-27 cells (6 × 10^6^ cells per mouse) using a two-sterile syringe. Starting on day 4 post-injection, tumor size was measured every 2 days, and the volume was measured by the following equation: 0.5 × long diameter × short diameter^2^. The mice were humanely euthanized on the day 18 post-injection. Subsequently, the tumor tissues were sectioned, paraffin-embedded, and then subjected to HE staining and immunohistochemistry (IHC). The study protocols were approved by the Experimental Animal Care and Ethics Committee of Hebei Medical University First Hospital (Approval No. S00825).

### 2.11 Immunohistochemistry (IHC)

Tissue samples from patients and nude mice were fixed in 4% paraformaldehyde (PFA) solution and paraffin-embedded using standard histological procedures (Shanghai YiYang Instrument Co., Ltd., China). Subsequently, 4-μm-thick sections were prepared and stained. For immunohistochemical analysis, the sections were processed using a Rabbit two-step detection kit (ZSGB-Bio, Beijing, China; Catalog number: PV-6001) in accordance with the manufacturer’s protocol. The following primary antibodies obtained from Proteintech (Wuhan, China) were used: INHBA (diluted 1:200; Proteintech; Catalog number: 17524-1-AP), Ki67 (diluted 1:100; Proteintech; Catalog number: 27309-1-AP), and ITGA6 (diluted 1:500; Proteintech; Catalog number: 27189-1-AP).

The IHC staining score included the proportion of positively stained tumor cells and staining intensity. The proportion of positively stained tumor cells was scored as follows: 0 (no tumor cells stained), 1 (<25% tumor cells stained), 2 (25%–50% tumor cells stained), 3 (50%–75% tumor cells stained), and 4 (75%–100% tumor cells stained). Staining intensity was graded using the following criteria: 3 (brown, strong staining), 2 (yellow brown, moderate staining), 1 (light yellow, weak staining), and 0 (no staining). The total staining score was calculated by multiplying the proportion of stained tumor cells by the staining intensity score. A score of ≤3 indicated negative expression, while a score of >3 indicated positive expression. Pathological diagnosis was made in accordance with the histological classification of tumors developed by the World Health Organization.

### 2.12 RNA Sequencing (RNA-Seq)

After 24-h transfection with siRNA-NC and siRNA-INHBA, total RNA was extracted from NUGC-3 cells. Beijing Novogene Technology Co., Ltd., performed RNA library preparation and sequencing analysis. Briefly, six samples (triplicates of siRNA negative control [siNC] and INHBA-targeting siRNA [siINHBA]) were sequenced on an Illumina NovaSeq 6000 platform. Raw sequencing data were subjected to quality control and filtering to obtain high-quality reads, which were then aligned with reference genomes using the HISAT2 (HISAT2 2.2.1) software for gene expression quantification. Differentially expressed genes (DEGs) were identified using the DESeq2 (DESeq2 v2.1) software with the threshold criteria of log2 |FoldChange| ≥ 0 and *p*-value < 0.05. INHBA silencing induced widespread transcriptome dysregulation, comprising 1112 and 846 up- and down-regulated genes, as shown in the gene expression heatmap and volcano plot. Through the analysis of RNA-seq-identified downregulated genes (log2Fold Change > 1, *p* < 0.05), along with the 3742 genes that are highly expressed in GC within the GEPIA2 database, several genes were identified as potential candidates for binding to the INHBA protein.

### 2.13 Co-Immunofluorescent (Co-IF) Assay

NUGC-3 cells were evenly seeded onto coverslips placed in six-well plates (7 × 10^5^ cells/well). At 20%–30% confluence, the cells were gently washed three times with 1× PBS (pH 7.2–7.4). Subsequently, the cells were sequentially processed as follows: blocked with 2% bovine serum albumin (BSA-V; Solarbio; Catalog number: A8020), fixed with 4% PFA, and penetrated with 0.2% Triton X-10 (Solarbio; Catalog number: T8200). Primary INHBA antibody (diluted 1:50; Proteintech; Catalog number: 17524-1-AP) and ITGA6 antibody (diluted 1:50; Santa Cruz Biotechnology, Dallas, TX, USA; Catalog number: sc-374057) were added to the cells and incubated overnight at 4°C. After incubation of the primary antibody, the cells were incubated with fluorescently labeled secondary antibodies (1:500 dilution; Cy3 Anti-Rabbit IgG, FITC Anti-Mouse IgG; Beyotime Biotechnology Co., Shanghai, China; Catalog numbers: A0516 and A0568) for 1 h at room temperature in the dark. Finally, nuclei were stained with 4^′^6^′^-diamino-2-phenylindole dihydrochloride (1 ug/mL) (Beyotime Biotechnology Co., Shanghai, China). Representative images were then acquired using a fluorescence microscope (Eclipse Boi, Nikon, Tokyo, Japan).

### 2.14 Co-Immunoprecipitation (Co-IP)

NUGC-3 cells were washed twice with 1× PBS (pH 7.2–7.4) under gentle conditions. Cell lysis was performed using the Pierce™ Classic Magnetic IP/Co-IP Kit (Thermo Scientific, Waltham, MA, USA; Cat# 88804) according to the manufacturer’s protocol. Then, the lysate was mixed with 10 μg of the INHBA, ITGA6, and IgG control antibodies obtained from Proteintech. Next, the mixture was incubated at room temperature for 2 h with gentle rotation. After the addition of magnetic beads to the lysate, the mixture was continuously rotated at room temperature for 1 h. After magnetic bead separation, proteins were eluted with an elution buffer. The pH was neutralized using a neutralization buffer. The resulting samples were analyzed via Western blot (WB).

### 2.15 Bioinformatics Analysis

The expression levels of INHBA mRNA in GC tissues and adjacent normal tissues were analyzed using the Gene Expression Omnibus database (GEO) (https://www.aclbi.com/static/index.html#/geo). Gene expression levels and survival analysis in GC were analyzed using Gene Expression Profiling Interactive Analysis 2 (GEPIA2) (http://gepia2.cancer-pku.cn/#index), whereas subcellular localization was analyzed using GeneCards (https://www.genecards.org/). Specific primers were designed and validated using PrimerBank (https://pga.mgh.harvard.edu/primerbank/) in combination with an advanced primer design tool (https://www.nc/bi.nlm.nih.gov/tools/primer-blast/). KEGG enrichment analysis was conducted on the DAVID (Database for Annotation, Visualization, and Integrated Discovery) (https://davidbioinformatics.nih.gov/). Several candidate genes regulated by INHBA are screened through the Venn diagram (https://bioinfogp.cnb.csic.es/tools/venny/).

### 2.16 Statistical Analysis

Three independent experiments were conducted for each group to ensure the reliability of the results, in accordance with established guidelines within the field. Normally distributed data were expressed as mean ± standard deviation, whereas non-normally distributed data were expressed as median and interquartile distance. All statistical analyses were conducted using SPSS 26.0 (IBM, Armonk, NY, USA) and GraphPad Prism 9.5 (GraphPad Software, La Jolla, CA, USA) software. Student’s *t*-test was employed for pairwise comparisons, one-way analysis of variance (ANOVA) for single-factor group comparisons, and two-way ANOVA for assessing interactions between two independent variables. *p* < 0.05 was considered statistically significant.

### 2.17 Data Availability

Data supporting the findings of this study are available from the corresponding author upon request. Gene Expression Profiling Interactive Analysis (GEPIA2) and Gene Expression Omnibus (GEO) at GSE63089 and GSE66229 provided the data used in this analysis.

## 3 Results

### 3.1 INHBA Is Upregulated in Patients with GC and Is Associated with Poor Prognosis

**Figure 1:**
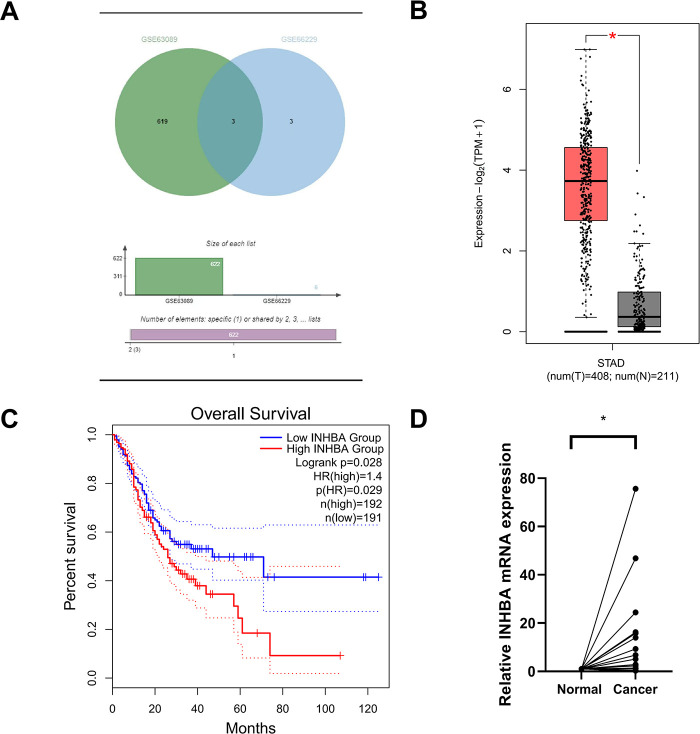

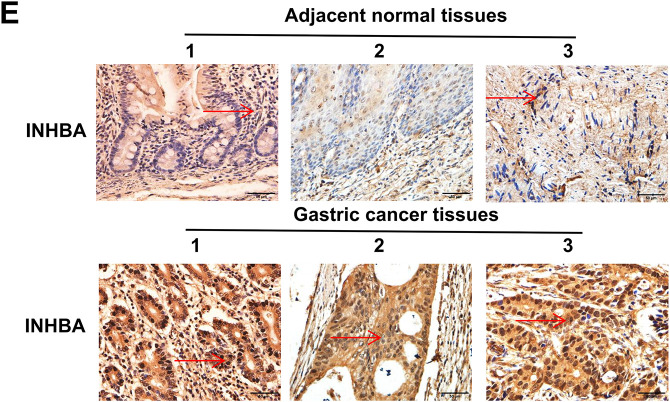
Increased INHBA expression is associated with poor prognosis in GC patients. (**A**) Expression of INHBA in the GEO database, including the GSE63089 and GSE66229 datasets. (**B**) INHBA is highly expressed in STAD tissues through the analysis of the online databases GEPIA2. (**C**) The survival analysis of INHBA expression levels and overall survival in patients with GC. (**D**) The expression levels of INHBA mRNA in 34 pairs of GC and adjacent normal tissue samples were detected by qRT-PCR. (**E**) Representative images showing INHBA protein expression levels in 50 pairs of GC and adjacent normal tissue samples, as detected by IHC. (magnification ×400). The red arrow indicates the INHBA expression area, which appears brown or yellow. *p < 0.05

To elucidate the role of INHBA in the GC pathophysiology, we initially assessed its expression in GC tissues and adjacent normal tissues using data from the GEO database, specifically the GSE63089 and GSE66229 datasets. Our analysis revealed substantial upregulation of INHBA expression in GC tissues relative to adjacent normal tissues, as presented in Fig. 1A. This finding was further corroborated by the significant upregulation of INHBA in stomach adenocarcinoma (STAD) tissues, as shown by analysis conducted using the GEPIA2 online tool (Fig. 1B). Survival analysis using GEPIA2 revealed that patients with GC demonstrating high INHBA expression had significantly poorer OS than those with low INHBA expression (Fig. 1C). Based on existing knowledge of the functional role of INHBA in GC, we used it as the target gene for further investigation. We verified INHBA mRNA expression in 34 pairs of GC tissues and their adjacent normal tissues via qRT–PCR. The results indicated a statistically significant upregulation of INHBA mRNA in GC tissues compared with normal tissues (*p* < 0.05) (Fig. 1D). To confirm these findings at the protein level, IHC was conducted on GC tissues and adjacent normal tissues, which revealed that INHBA protein was localized in the cytoplasm of GC cells and significantly overexpressed in GC tissues (*p* < 0.001, *χ*^*2*^ = 19.485) (Fig. 1E, Table 1). Furthermore, the correlation between INHBA expression and the clinical characteristics of patients with GC was investigated. Our analysis revealed that the INHBA expression was not associated with gender, age, tumor size, and tumor differentiation. However, it was found to be significantly associated with tumor lesion, lymph node metastasis, and TNM stage (*p < 0.05, **p < 0.01, ***p < 0.001) (Table 2). These findings suggest that INHBA overexpression is closely related to poor prognosis in patients with GC, indicating its potential as a prognostic biomarker and a therapeutic target in GC. Finally, the mRNA and protein expression levels of INHBA in different GC cell lines and GES-1 cells were evaluated via qRT-PCR and WB (Supplementary Fig. S1A,B).

**Table 1:** The expression level of INHBA protein in two groups

Groups	Cases	INHBA Expression		Positive Rate (%)	χ^2^	*p*
					Positive	Negative
Gastric cancer tissues	50	38	12	76%	19.485	<0.001***
Adjacent normal tissues	50	16	34	32%		

Note: INHBA, Inhibin subunit beta A; ***p < 0.001.

**Table 2:** The relationship between the expression of INHBA in gastric cancer tissue and the clinical and pathological data of patients

Groups	Cases	INHBA Expression		χ^2^	*p*
				Positive	Negative
**Sex**					
Male Female	38 12	29 9	9 3	0.009	0.926
**Age (years)**					
≤60	8	6	2	0.005	0.942
>60	42	32	10		
**Tumor Lesion**					
Cardia	8	2	6	13.581	<0.001***
Non-cardia	42	36	6		
**Tumor size**					
≤4 cm	22	18	4	0.729	0.393
>4 cm	28	20	8		
**Differentiation**					
Poorly	36	28	8	0.223	0.637
Well	14	10	4		
**Lymph node metastasis**					
Absent	11	5	6	7.214	0.007**
Present	39	33	6		
**TNM staging**					
I	1	0	1	10.147	0.017*
II	13	7	6		
III	26	21	5		
IV	10	10	0		

Note: TNM, tumor node metastasis; INHBA, Inhibin subunit beta A; *p < 0.05, **p < 0.01, ***p < 0.001.

### 3.2 INHBA Knockdown Suppresses the Proliferation, Migration, and Invasion of GC Cells and Promotes Apoptosis In Vitro

**Figure 2:**
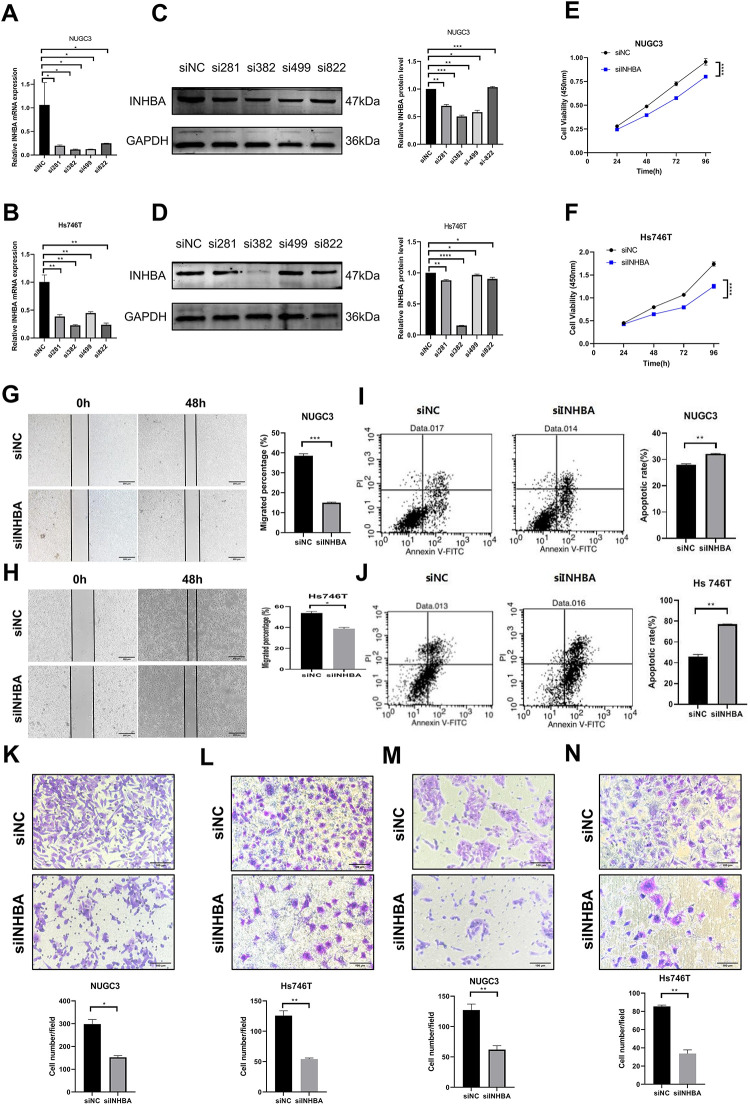
Knockdown of INHBA in GC cells suppresses cell proliferation, migration and invasion *in vitro*. (**A**,**B**) The expression of INHBA mRNA in NUGC-3 and Hs746T cells was detected by qRT-PCR. (**C**,**D**) The expression of INHBA protein in NUGC-3 and Hs746T was detected by WB. (**E**,**F**) The effect of INHBA knockdown on the proliferation of NUGC-3 and Hs746T cells was determined by the CCK-8 assay. (**G**,**H**) The effect of INHBA knockdown on the migration of NUGC-3 and Hs746T cells was assessed by the wound healing assay. Scale bar, 200 μm. (**I**,**J**) The effect of INHBA knockdown on the apoptosis of NUGC-3 and Hs746T cells was determined by flow cytometry. (**K**,**L**) The effect of INHBA knockdown on the migration of NUGC-3 and Hs746T cells was evaluated using the Transwell migration assay. Scale bar, 100 μm. (**M**,**N**) The effect of INHBA knockdown on the invasion of NUGC-3 and Hs746T was assessed using the Transwell invasion assay. Scale bar, 100 μm. Data are presented as means ± SD. *p < 0.05, **p < 0.01, ***p < 0.001, ****p < 0.0001

To explore the functional role of INHBA in GC, we first downregulated INHBA expression in NUGC-3 and Hs746T cell lines and evaluated its impact on various cellular processes. After transfection with siINHBA, considerable reductions in INHBA mRNA (Fig. 2A,B) and protein (Fig. 2C,D) levels were observed compared with the siNC. Among the four siRNA sequences of INHBA (si281, si382, si499, si822), the si382 decline elicited the most evident effect; thus, it was selected for subsequent cell function knockdown experiments. The CCK-8 assay revealed that the viability of NUGC-3 and Hs746T cells was markedly reduced after INHBA knockdown compared with the control group (Fig. 2E,F). The wound healing assay revealed that the migratory capacity of INHBA knockdown cells was significantly impaired compared with that of the control group in NUGC-3 and Hs746T cells after 48 h (Fig. 2G,H). Flow cytometry analysis revealed a considerable increase in the number of apoptotic cells in NUGC-3 and Hs746T cell lines after INHBA silencing compared with that in the control group (Fig. 2I,J). The Transwell assay results indicated a significant reduction in the migratory and invasive capacities of NUGC-3 and Hs746T cell lines in the INHBA-KO group (Fig. 2K–N). These findings suggest that INHBA downregulation promotes apoptosis while inhibiting the invasion, migration, and proliferation of GC cells.

### 3.3 Overexpression of INHBA Promotes Proliferation, Migration, and Invasion of GC Cells and Suppresses Apoptosis In Vitro

**Figure 3:**
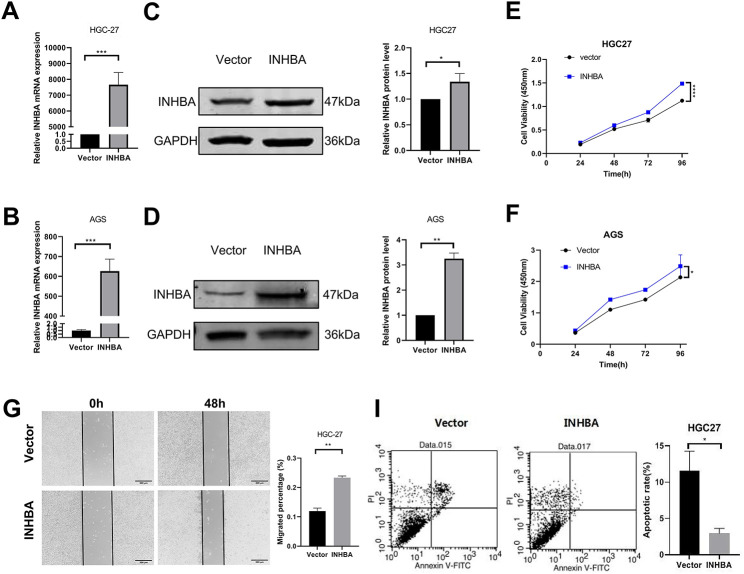

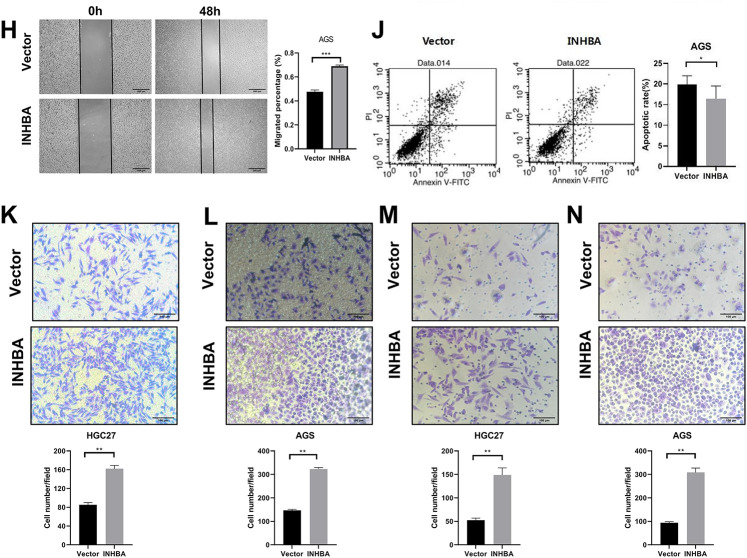
Overexpression of INHBA in GC cells and human gastric mucosa. Cells promotes cell proliferation, migration and invasion *in vitro*. (**A**,**B**) The expression of INHBA mRNA in HGC-27 and AGS was detected by qRT-PCR. (**C**,**D**) The expression of INHBA protein in HGC-27 and AGS was detected by WB. (**E**,**F**). The effect of INHBA overexpression on the proliferation of HGC-27 and AGS cells was assessed using the CCK-8 assay. (**G**,**H**) The effect of INHBA overexpression on the migration of HGC-27 and AGS cells was evaluated through a wound healing assay. Scale bar, 200 μm. (**I**,**J**) The effect of INHBA overexpression on the apoptosis of HGC-27 and AGS was analyzed by flow cytometry. (**K**,**L**) The effect of INHBA overexpression on the migration of HGC-27 and AGS cells was determined utilizing a Transwell migration assay. Scale bar, 100 μm. (**M**,**N**). The effect of INHBA overexpression on the invasion of HGC-27 and AGS cells was assessed via a Transwell invasion assay. Scale bar, 100 μm. Data are presented as means ± SD. *p < 0.05, **p < 0.01, ***p < 0.001, ****p < 0.0001

To further investigate the function of INHBA in cellular biological processes, we upregulated its expression in HGC-27 and AGS cell lines. pcDNA3.1-INHBA transfection considerably increased the INHBA mRNA (Fig. 3A,B) and protein (Fig. 3C,D) levels compared with pcDNA3.1-vector control transfection. The CCK-8 assay demonstrated that INHBA overexpression significantly increased HGC-27 and AGS cell viability compared with the control group (Fig. 3E,F). The wound-healing assay revealed that INHBA upregulation significantly enhanced cell migration in HGC-27 and AGS cells after 48 h (Fig. 3G,H). Meanwhile, flow cytometry analysis showed that INHBA upregulation significantly alleviated apoptosis in HGC-27 and AGS cells (Fig. 3I,J). Moreover, the Transwell assay demonstrated that INHBA upregulation significantly enhanced the migratory and invasive capacities of HGC-27 and AGS cells (Fig. 3K–N). In summary, our findings suggest that INHBA acts as a pro-oncogene in GC, promoting invasion, migration, and proliferation while inhibiting apoptosis.

### 3.4 INHBA Overexpression Promotes GC Growth In Vivo

**Figure 4:**
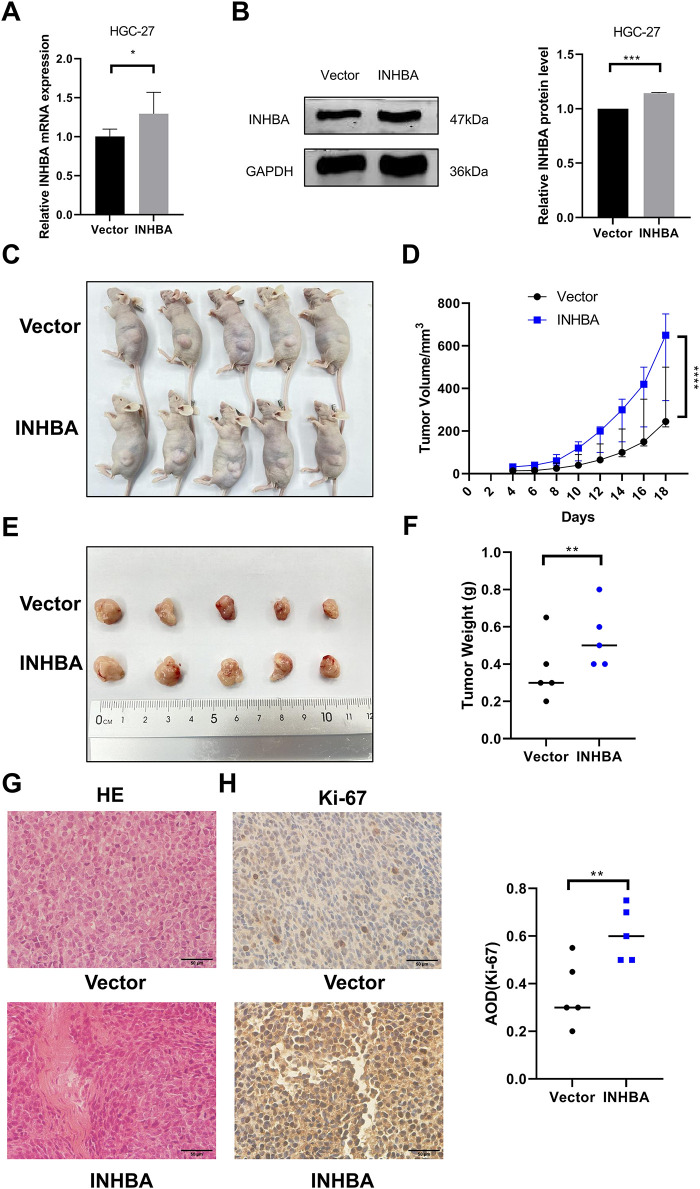
INHBA promotes the growth of GC *in vivo*. (**A**) Verification of stable bead over-expression efficiency was detected by qRT-PCR. (**B**) Verification of stable bead over-expression efficiency was detected by WB. (**C**) subcutaneous xenograft models. (**D**) Tumor growth curve. (**E**,**F**) Images (**E**) and weight (**F**) of subcutaneous xenograft models were analyzed. (**G**) Representative images of tumor tissue sections stained with HE. (magnification ×400). (**H**) Representative image of Ki67 IHC staining in tumor tissue sections. (magnification ×400). Data are shown as means ± SD.*p < 0.05, **p < 0.01, ***p < 0.001, ****p < 0.0001

To better investigate the role of INHBA *in vivo*, we created a subcutaneous xenograft model in BALB/C nude mice using HGC-27 cells transfected with INHBA-overexpressing or control vectors. Through qRT-PCR and WB, we first confirmed INHBA overexpression in stably transfected cell lines (Fig. 4A,B), validating the successful construction. HGC-27 cells stably transfected with INHBA-overexpressing or empty vectors were subcutaneously injected into BALB/C nude mice to construct a subcutaneous xenograft model. Fig. 4C–F demonstrate that INHBA-overexpressing tumors displayed significantly higher growth rates, volumes, and weights than the vector group in subcutaneous xenograft models. Histopathological analysis of subcutaneous xenograft model tissues via HE staining revealed that the cellular architecture and morphological features closely resembled those of human GC tissues (Fig. 4G). IHC for Ki67 revealed a considerable increase in the cell proliferative capacity of the INHBA-overexpressing group than the vector group (Fig. 4H). These findings indicate that INHBA overexpression promotes GC progression *in vivo*.

### 3.5 ITGA6 Is a Potential Target of INHBA in GC

**Figure 5:**
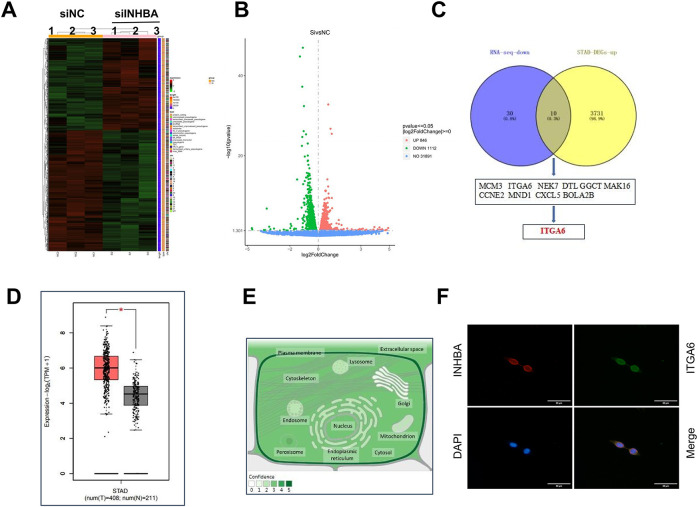

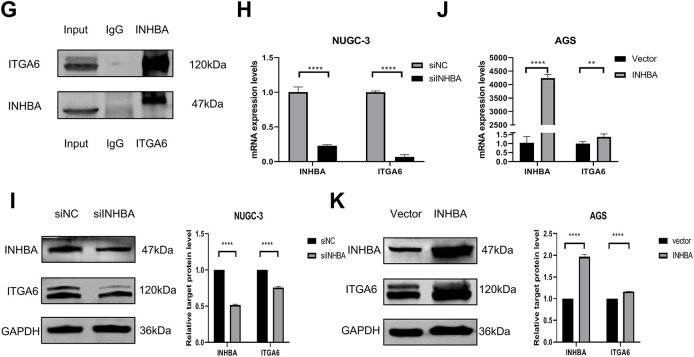
ITGA6 is the regulatory target of INHBA. (**A**) Differential expression genes (DEGs) heat map identified by RNA-seq. (**B**) The number of DEGs identified by RNA-seq. (**C**) Overlap analysis of RNA-seq and STAD-DEGs-up identified genes. (**D**) Expression of ITGA6 in GC paired tissue cohort from the GEPIA2 database. (**E**) Subcellular localization of ITGA6 from GeneCards. (**F**,**G**) The interaction between INHBA and ITGA6 was verified by Co-IF (**F**) and Co-IP (**G**) experiments. Scale bar, 50 μm. (**H**) qRT-PCR was used to detect ITGA6 mRNA expression after INHBA knockdown in NUGC-3 cells. (**I**) WB was used to detect ITGA6 protein expression after INHBA knockdown in NUGC-3 cells. (**J**) qRT-PCR was used to detect ITGA6 mRNA expression after INHBA overexpression in AGS cells. (**K**) WB was used to detect ITGA6 protein expression after INHBA overexpression in AGS cells. Data are presented as means ± SD. *p < 0.05, **p < 0.01, ****p < 0.0001

To elucidate the pathophysiological role of INHBA in GC, we performed transcriptome RNA-seq on NUGC-3 cells transfected with either nontargeting siRNA (siNC) or siINHBA. The results indicated that INHBA silencing induced widespread transcriptome dysregulation, with 1958 differentially expressed genes identified, comprising 1112 and 846 up- and down-regulated genes, respectively, as shown in the gene expression heatmap and volcano plot (Fig. 5A,B). Through the analysis of RNA-seq-identified downregulated genes (log2Fold Change > 1, *p* < 0.05), along with the 3742 genes that are highly expressed in GC within the GEPIA2 database, 10 genes were identified as potential candidates for binding to the INHBA protein (Fig. 5C). As INHBA is localized extracellularly and cytoplasmic, we selected membrane proteins from the candidate genes as potential targets for INHBA regulation. Among the 10 candidate genes, ITGA6 was the only membrane protein that exhibited broad distribution across multiple cellular compartments in GC cells. Bioinformatics analysis using the GEPIA2 database revealed that ITGA6 showed a constitutive overexpression in gastric adenocarcinoma specimens (Fig. 5D), with multicompartment localization patterns demonstrating predominant distribution in the cytomembrane, cytoplasm, and nucleus domains (Fig. 5E). Therefore, we speculated that ITGA6 is a potential downstream target gene. Subsequently, Co-IF and Co-IP assays were conducted to demonstrate the interaction between INHBA and ITGA6. Immunofluorescence co-localization analysis revealed that co-localization of INHBA and ITGA6 is mainly observed in the cytoplasm and cytomembrane of GC cells (Fig. 5F). Co-IP assay confirmed direct interaction between INHBA and ITGA6 in GC cells (Fig. 5G). To confirm the INHBA-mediated regulation of ITGA6 expression, we performed bidirectional INHBA modulation (knockdown and overexpression) in GC cells. INHBA knockdown decreased ITGA6 mRNA and protein expressions in NUGC-3 cells (Fig. 5H,I). Conversely, INHBA upregulation increased the ITGA6 mRNA and protein expression levels in AGS cells (Fig. 5J,K). These results suggest that INHBA regulates downstream gene expression at the transcriptional and translational levels. In summary, these results indicated a functional interaction between INHBA and ITGA6 in GC and that ITGA6 may act as a potential modification target of INHBA in GC.

### 3.6 ITGA6 Is Highly Expressed in GC and Interacts with INHBA

Next, we evaluated ITGA6 expression in GC cell lines and clinical specimens.

**Figure 6:**
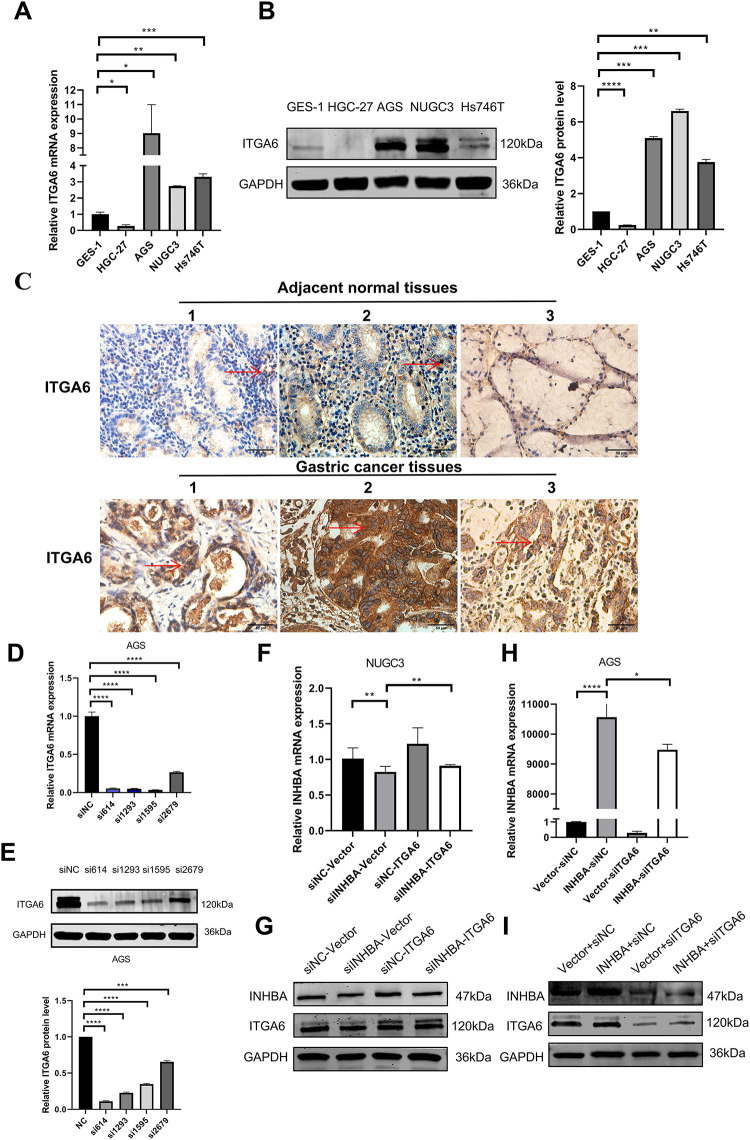
Expression of ITGA6 and its interaction with INHBA in GC. (**A**) The basal expression of ITGA6 mRNA in GC cell lines and GES-1 was detected by qRT-PCR. (**B**) The basal expression of ITGA6 protein in cell lines was detected by WB. (**C**) The expression of ITGA6 protein in 30 pairs of GC paired tissues was detected by IHC. (magnification ×400). The red arrow indicates the ITGA6 expression area, which appears brown or yellow. (**D,E**) Four siRNA-ITGA6 sequences were transfected simultaneously into AGS cells. (**F,G**) The relative expression level of INHBA mRNA and protein was detected by qRT-PCR and WB after instantaneous co-transfection of INHBA siRNA and ITGA6 overexpression plasmid in NUGC-3. (**H,I**) The relative expression level of INHBA mRNA and protein were detected by qRT-PCR and WB after instantaneous co-transfection of ITGA6 siRNA and INHBA overexpression plasmid in AGS. Data are presented as means ± SD. *p < 0.05, **p < 0.01, ***p < 0.001, ****p < 0.0001

Quantitative analysis revealed that the majority of GC cell lines exhibited a significant increase in ITGA6 mRNA expression compared with normal gastric mucosa cells (Fig. 6A). WB analysis revealed that NUGC-3 exhibited the highest ITGA6 protein expression among all the tested cell lines (Fig. 6B). IHC analysis of 30 paired clinical specimens revealed that the GC tissues exhibited higher ITGA6 expression (positive rate 80%) than the adjacent normal tissues (positive rate 40%) (Fig. 6C). These results suggest that ITGA6 is highly expressed in GC tissues and various GC cell lines. Further studies are warranted to determine how the INHBA–ITGA6 interaction contributes to gastric carcinogenesis. The cell lines were selected based on the baseline ITGA6/INHBA expression level. Functional screening of four ITGA6-targeting siRNAs in AGS cells with the highest ITGA6 mRNA expression revealed that siITGA6-614 showed superior silencing efficiency and was thus prioritized for further analyses (Fig. 6D,E). Subsequently, we applied reciprocal modulation strategies in GC cell models: NUGC-3 received ITGA6 overexpression (pcDNA3.1-ITGA6) and depletion with INHBA (siRNA-INHBA), whereas AGS underwent ITGA6 silencing (siRNA-ITGA6) and INHBA overexpression (pcDNA3.1-INHBA). qRT–PCR and WB were employed to identify variations in INHBA expression between the groups. The results indicated that ITGA6 overexpression could inhibit the INHBA knockdown effect in NUGC-3 cells (Fig. 6F,G). Similarly, ITGA6 silencing mitigated the effect of INHBA overexpression in AGS cells (Fig. 6H,I). These results indicate that ITGA6 can influence INHBA expression in GC cells.

### 3.7 INHBA Targets ITGA6 to Activate the MAPK Signaling Pathway in GC Progression

**Figure 7:**
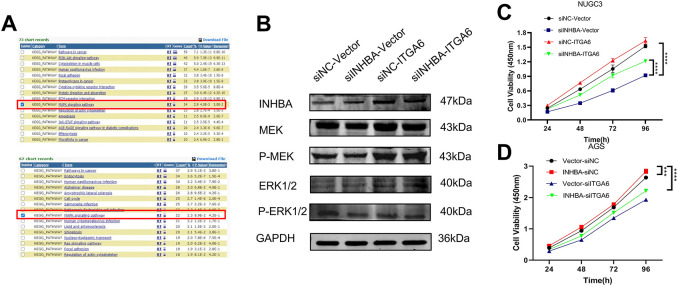

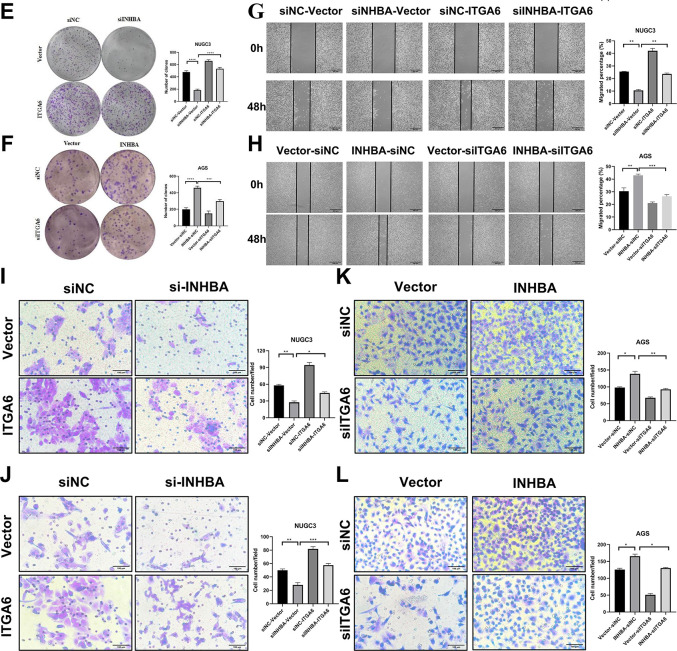
Oncogenic function of INHBA depends on ITGA6 and the MAPK signaling pathway. (**A**) KEGG enrichment result of the differentially expressed gene sets from the GEPIA2 database. (**B**) The expression levels of MAPK signaling pathway-related proteins were detected by WB after instantaneous co-transfection of INHBA siRNA and ITGA6 overexpression plasmid in NUGC-3. (**C,E,G,I,J**) The effects of ITGA6 on INHBA in NUGC-3 cells were detected by CCK-8 assay (**C**), colony formation assay (**E**), wound healing assay (**G**), Scale bar, 200 μm, Transwell migration assay (**I**) and Transwell invasion assay (**J**). Scale bar, 100 μm. (**D,F,H,K,L**) The effects of ITGA6 on INHBA in AGS cells were detected by CCK-8 assay (**D**), colony formation assay (**F**), wound healing assay (**H**), Scale bar, 200 μm, Transwell migration assay (**K**) and Transwell invasion assay (**L**). Scale bar, 100 μm. Data are presented as means ± SD. *p < 0.05, **p < 0.01, ***p < 0.001, ****p < 0.0001

To further explore the molecular mechanism of INHBA and ITGA6, we selected the top 1000 genes co-expressed with INHBA and ITGA6 in GC from the GEPIA2 database and analyzed the signaling pathways these genes controlled. KEGG pathway enrichment analysis using the DAVID revealed that gene clusters were markedly enriched in the MAPK signaling pathways (Fig. 7A). Therefore, we speculated that INHBA activates ITGA6 via the MAPK signaling pathway to promote GC progression. Subsequently, we examined the ability of ITGA6 to rescue the suppression of the MAPK signaling pathway induced by INHBA knockdown. WB analysis revealed that INHBA silencing induced concomitant downregulation of MAPK cascade components (p-MEK, p-ERK1/2) in NUGC-3 cells. Simultaneously, ITGA6 overexpression rescued the suppression of MAPK phosphoproteins, such as p-MEK and p-ERK1/2, induced by INHBA knockdown (Fig. 7B). These results suggest that INHBA regulates the activation of the MAPK signaling pathway via ITGA6. Functional interaction studies demonstrated that ITGA6 modulates INHBA-induced oncogenic phenotypes. Functional rescue assays revealed that ITGA6 overexpression rescued the attenuation of cell proliferation, migration, and invasion in NUGC-3 cells induced by INHBA silencing (Fig. 7C,E,G,I,J). Similarly, downregulation of the ITGA6 expression reduced the enhancement of proliferation, migration, and invasion in AGS cells caused by INHBA overexpression (Fig. 7D,F,H,K,L). In conclusion, the INHBA/ITGA6 axis promotes gastric carcinogenesis by activating the MAPK signaling pathway, with ITGA6 acting as the indispensable mediator of INHBA oncogenic activity. In summary, elevated INHBA levels increased the expression of ITGA6 mRNA and protein, which further activated the MAPK signaling pathway and eventually contributed to GC progression.

## 4 Discussion

This study demonstrated that INHBA overexpression is correlated with poor prognosis in patients with GC. Functional validation using a series of assays showed that INHBA contributed to tumorigenic phenotypes *in vitro* and *in vivo*. INHBA knockdown suppresses the proliferation, migration, and invasion of GC cells and promotes apoptosis, contrasting with the oncogenic effects of INHBA overexpression. Mechanistically, INHBA promotes gastric carcinogenesis by upregulating ITGA6, thereby activating the MAPK signaling pathway to promote tumor progression.

Previous studies have demonstrated that GC tissues exhibited higher INHBA expression than the adjacent normal tissues. These results were significantly associated with lymph node metastasis and TNM staging [18]. In this study, the clinical and pathological characteristics of 50 pairs of GC and adjacent normal tissues were evaluated, and the results were consistent with those of previous studies. Notably, the results of our study indicated that the positive expression rate of INHBA was considerably lower in cardia cancer than in noncardia cancer. Further in-depth and comprehensive research is warranted to elucidate the clinical significance of this finding in guiding treatment decisions for cardia and noncardia cancers. In conclusion, INHBA may be a clinically useful and prognostic indicator of poor patient survival.

The experimental results indicated that INHBA silencing markedly inhibited the proliferation, migration, and invasion of GC cells while promoting apoptosis, confirming the functional influence of INHBA on GC cellular processes. In contrast, INHBA overexpression elicited opposite effects. Furthermore, animal studies using immunodeficient nude mice reported that INHBA overexpression induced the progression of gastric tumors. Previous studies have shown that INHBA overexpression enhances malignant behaviors in GC cell *in vitro* models, whereas the genetic inhibition of INHBA suppresses these oncogenic phenotypes [14,16]. Several studies have reported that INHBA is closely associated with various types of cancer, such as esophageal squamous cancer [7], colorectal cancer [19], cervical cancer [20], and ovarian cancer [21]. Consistent with our data, these results support the role of INHBA in promoting tumorigenic properties (proliferation and metastasis) during gastric carcinogenesis.

We sought to identify the genes targeted by INHBA to elucidate its role in the biological mechanisms underlying GC pathogenesis. Integrated analysis of downregulated genes in RNA-seq data, STAD- DEGs-up overlap, database screening, and literature viewing identified ITGA6 as a candidate gene. WB analysis following Co-IP and Co-IF experiments demonstrated a physical interaction between INHBA and ITGA6 in GC cells, which is a novel discovery within the field. As a member of the integrin family, ITGA6, also known as α6-integrin or CD49f, is a cell-surface protein that mediates cell-to-cell and cell-to-stroma adhesion, which is vital to cell proliferation, migration, survival, and differentiation [22]. ITGA6 has been shown to play a crucial role in ovarian, bladder, and pancreatic cancers by several previous studies [23–25]. These data are consistent with our ITGA6 observations in GC, as shown in Fig. 6A–C. To investigate how INHBA modulates ITGA6 in GC cell lines, we demonstrated that INHBA regulates ITGA6 expression at both the transcriptional and translational levels, as shown in Fig. 5H–K. In summary, INHBA may promote GC progression by enhancing ITGA6 expression through mRNA and protein binding.

KEGG pathway enrichment analysis using the DAVID revealed that gene clusters co-expressed with INHBA and ITGA6 were significantly enriched in the MAPK signaling pathway. The MAPK signaling pathway plays a pivotal role in transmitting extracellular signals to intracellular effectors, and its cascade dynamics are frequently dysregulated by epigenetic modifications in diverse pathological contexts, particularly in oncogenic transformation. Studies have shown that the MAPK pathway is vital to the process of GC development, including cellular proliferation, invasion, migration, and metastasis [26]. Previous studies have reported that MAPK signaling pathway-related proteins are widely distributed in various types of cancer, such as colorectal cancer [27], esophageal squamous cancer [28], pancreatic cancer [29], and hepatocellular carcinoma [30]. MEK (mitogen-activated protein kinase) is a key kinase in the MAPK signaling pathway that can be activated by upstream kinases and then phosphorylated and activated downstream (extracellular signal-regulated protein kinase) to regulate cell growth, differentiation, and proliferation. Therefore, we hypothesized that INHBA could activate the MAPK signaling pathway to promote GC progression. We found that suppression of MAPK pathway activation induced by INHBA knockdown was reversed by ITGA6 overexpression which directly activated the MAPK signaling pathway. Rescue studies have shown that the INHBA–ITGA6–MAPK axis critically regulates the malignant behaviors of GC cells, including proliferation, migration, and invasion. These findings suggest that the INHBA–ITGA6–MAPK axis promotes GC progression, suggesting a potential and promising therapeutic target.

However, this study has some limitations requiring future investigation. Although mechanistically confined to the MAPK signaling pathway, our findings do not exclude the potential involvement of other pathways in INHBA-driven oncogenic processes. The specific mechanisms and interactions of these signaling pathways need to be further explored. Although this study established the therapeutic potential of INHBA/MAPK/ITGA6 axis inhibition in gastric cells, pharmacological validation using small-molecule inhibitors requires preclinical assessments of efficacy *in vitro* and *in vivo* models. ERK inhibitors have been applied in the targeted therapy of some tumors [31]. In the future, some MAPK (ERK) inhibitors are expected to be applied to patient-derived organoids and some GC patients may benefit from these findings in their potential treatment.

Liquid biopsies typically comprise the sampling of the peripheral blood and are attractive since they are less invasive than surgical tumour tissue biopsies. The latest research on liquid biopsies for disease diagnosis, assessing treatment efficacy, relapse, and recurrence in neuroblastoma, was promising [32]. Given that our study identifies the INHBA/ITGA6/MAPK axis, if INHBA or ITGA6 can be detected in circulating tumour DNA and circulating tumour RNA released by GC patients, a potential application of liquid biopsies should facilitate their implementation in GC clinical practice.

## 5 Conclusion

In conclusion, this study demonstrated that INHBA plays a pivotal oncogenic role in promoting GC cell proliferation, migration, and invasion. It also elucidated the molecular pathogenesis of INHBA via the ITGA6 interaction-mediated MAPK signaling pathway. This study identified INHBA as a potential diagnostic and prognostic biomarker. Furthermore, it showed that the INHBA/ITGA6 axis affects GC development by regulating the MAPK signaling pathway, which is emerging as a druggable target, providing a molecular rationale to support GC precision therapy.

## Supplementary Materials





## Data Availability

The data that support the findings of this study are available from the Corresponding Authors, [Jia Wang and Weifang Yu], upon reasonable request.

## Supplementary Materials

The supplementary material is available online at https://www.techscience.com/doi/10.32604/or.2025.070333/s1.
