# Supplementary material for: INHBA Promotes the Progression of Gastric Cancer by Activating MAPK Signaling Pathway via Targeting ITGA6
Source: Oncol Res. 2026 Feb 24;34(3):25. doi: 10.32604/or.2025.070333 (PMC12963699; doi:10.32604/or.2025.070333)
Supplement: Supplementary file 1 [file OncolRes-34-70333-s001.docx]

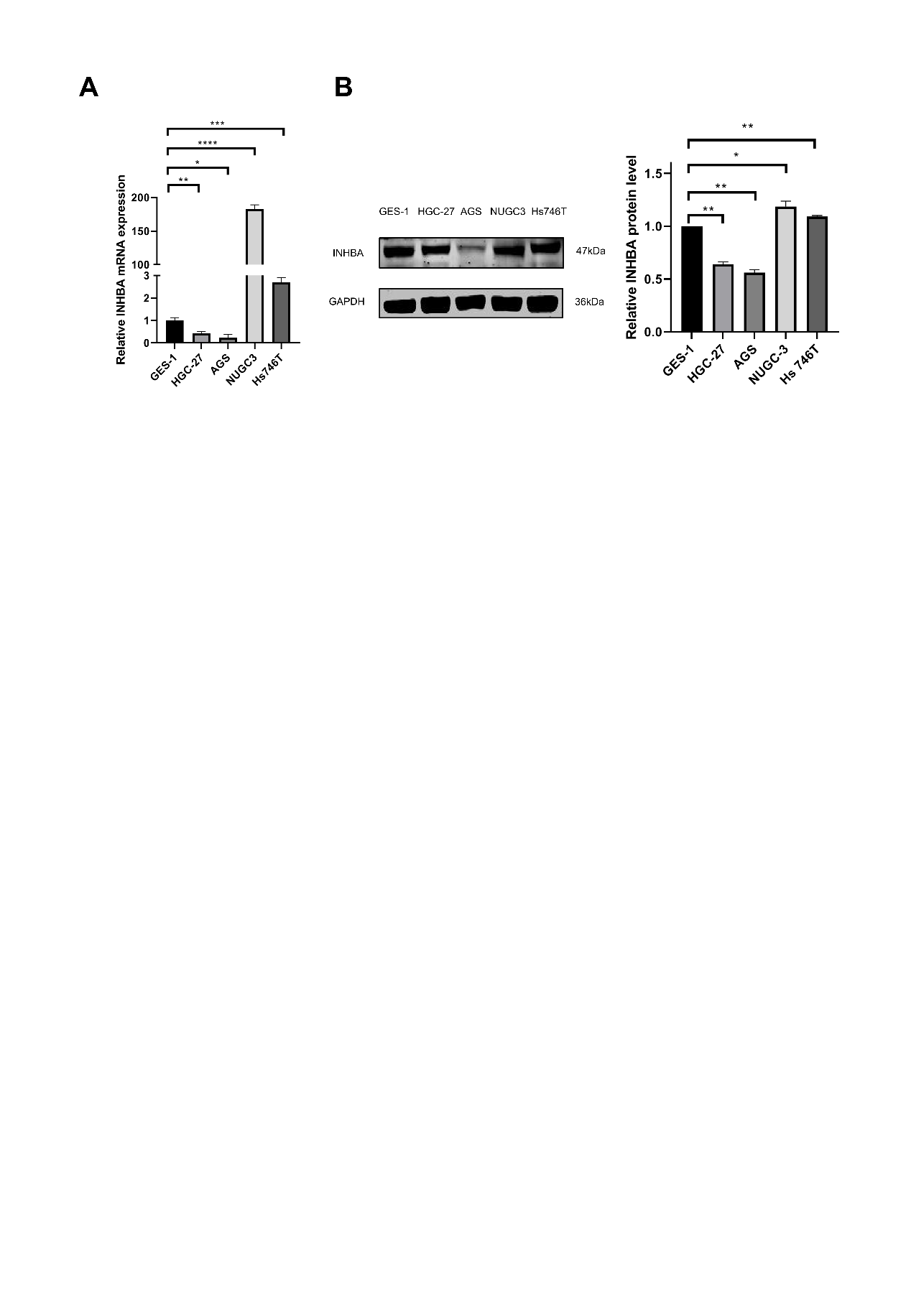


**Supplementary Figure S1.** **Levels of INHBA mRNA and protein in GC cell lines and GES-1.** (A) The expression of INHBA mRNA in GC cell lines and GES-1 was detected by qRT-PCR. (B) The basal expression of INHBA protein in GC cell lines and GES-1 cell lines was detected by WB. Data are presented as means±SD. Compared with GES-1, INHBA expression was lower in HGC-27 and AGS cells, but higher in NUGC-3 and Hs 746T cells. Therefore, for subsequent experiments, we selected HGC-27 and AGS for INHBA overexpression, chose NUGC-3 and Hs 746T for INHBA knockdown. **p* <0.05, ***p* <0.01, ****p* <0.001, *****p*<0.0001

**Supplementary Table S1. The primer sequences of INHBA, ITGA6, β-actin.**

| Primers | Forward primers 5’-3’ | Reverse primers 5’-3’ |
| --- | --- | --- |
| INHBA | CATTGCTCCCTCTGGCTATCAT | GCACACAGCACGATTTGAGGTT |
| ITGA6 | CACATCTCCTCCCTGAGCAC | TATCTTGCCACCCATCCTTG |
| β-actin | GAGTCAACGGATTTGGTCGT | CATGGGTGGAATCATATTGGA |
